# Rationalizing Spin-Crossover Properties of Substituted Fe (II) Complexes

**DOI:** 10.1021/acs.inorgchem.5c01523

**Published:** 2025-07-23

**Authors:** Gerard Comas-Vilà, Pedro Salvador

**Affiliations:** Institut de Química Computacional i Catàlisi i Departament de Química of Computational Chemistry and Catalysis, Chemistry Department, 16738 University of Girona, Montilivi Campus, Girona, Catalonia 17003, Spain

## Abstract

We investigate spin-state transitions in a series of 24 [Fe^II^(bpp^X^)_2_]^2+^ spin-crossover (SCO) complexes using density functional theory (DFT). The TPSSh/def2-TZVP approach demonstrates reasonable accuracy in predicting spin-state energetics compared to other functionals, though significant deviations persist in transition temperature (*T*_1/2_) estimates. Temperature-dependent and quasi-harmonic corrections for low-frequency vibrational contributions to enthalpic and entropic terms yielded only marginal improvements. To improve *T*_1/2_ prediction accuracy, we develop electronic descriptors based on effective fragment orbitals (EFOs) and their occupations, quantifying ligand σ-donation and π-acceptor characteristics that govern ligand field strength. Additionally, we introduce a resonance descriptor (*R*) derived solely from the effective atomic orbitals (eff-AOs) of isolated ligands. Our analysis reveals that electron-donating groups (EDGs) enhance π-electron density in the ligands while simultaneously reducing both σ-donor and π-acceptor capabilities, ultimately lowering the *T*_1/2_ value. These descriptors perform reasonably well also for a set of 12 [Fe^II^(pybox^X^)_2_]^2+^ SCO complexes. This new methodology provides a computationally efficient framework for modulating spin-state properties in transition metal complexes, enabling rational design of SCO materials.

## Graphical abstract

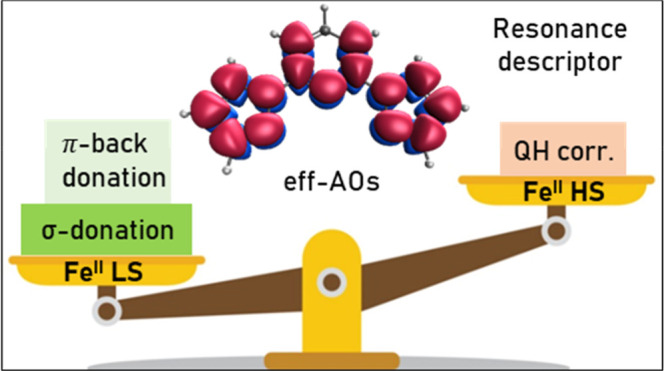

## Introduction

Transition metal (TM) complexes with d^4^–d^7^ electron configuration may have two or more accessible spin states. When external heat is applied, these complexes can undergo a reversible transition, resulting in the spin-crossover (SCO) phenomenon^1^ affecting the material’s physical and optical properties, including the magnetic moment, color, and conductivity.^2,3^ While this behavior has been seen in biological systems with iron-based porphyrins,^4^ SCO complexes have recently been extensively studied and have garnered attention due to their potential applications in molecular materials and nanoscience as molecular switches.^5–8^

The phenomenon of SCO was first discovered by Cambi and Szegö in 1931 for Fe­(III) compounds.^9^ Since then, many other first-row transition metal complexes, including Mn­(II/III),^10,11^ Fe­(II/III),^12–17^ Co­(II/III)^18,19^ and Ni­(II)^20,21^ have been studied. Among these, the most widely studied are octahedral Fe­(II) SCO complexes with a d^6^ electronic configuration and nitrogen-donor ligands. This is due to the pronounced differences in electronic and geometric properties between the low-spin (LS) and high-spin (HS) states. For Fe­(II) ion, the number of unpaired electrons shifts from zero in the diamagnetic LS state (*S* = 0) to four in the paramagnetic HS state (*S* = 2), leading to a dramatic magnetic transition.

A thermally induced LS → HS transition requires the free energy difference (Δ*G*) between the two states to be small, typically within ∼5 kcal·mol^–1^.^22^ This equilibrium is determined by the balance of enthalpic (Δ*H*) and entropic (Δ*S*) contributions, both of which depend on the metal center and its ligand field. Since spin crossover is entropy-driven, increasing the temperature favors the HS state by compensating for its higher enthalpy. The entropic stabilization (*T*Δ*S*) primarily results from the HS state’s greater spin multiplicity, which increases electronic degeneracy, as well as its longer metal–ligand bonds due to population of antibonding e_g_ orbitals, leading to enhanced vibrational entropy.

The temperature at which the LS and HS are equally populated is called the transition temperature (*T*_1/2_), an important parameter for characterizing the SCO compounds. Experimentally tuning the properties of SCO molecules remains a significant challenge, making it difficult to design compounds with targeted characteristics prior to synthesis. To overcome this limitation, theoretical methods have emerged as valuable tools for elucidating structure–property relationships and guiding experimental efforts. Over time, two complementary computational strategies have been developed to study SCO systems. The first approach focuses on direct prediction of *T*_1/2_ values through electronic structure calculations of both spin states (vide infra). Correlated methods, in particular CASPT2 ^23^ and NEVPT2,^24^ have been explored for spin-state splitting energies for some Fe and Co complexes. However, these methods tend to overstabilize high-spin states.^25–28^ Consequently, it has been concluded that more accurate methods must be used. To address this concern, a benchmark study was conducted based on experimental data from 17 first-row TM complexes.^29^ Among all the methods evaluated, the single-reference coupled cluster (CC) with singles, doubles, and perturbative triples CCSD­(T) method was identified as the most accurate, achieving a MAE of 1.5 kcal mol^–1^. Furthermore, a computationally efficient protocol was developed to approximate the CCSD­(T)/CBS (complete basis set) limit of TM spin state energetics in 13 chemically diverse complexes.^30^ The protocol utilized the explicitly correlated CCSD­(T#)-F12a approximation, which features a modified scaling of the perturbative triples term, denoted as (T#). The proposed computational protocol demonstrated the ability to reproduce the reference CCSD­(T)/CBS spin state energetics within a mean deviation of 0.2 kcal/mol. Additionally, a domain-based pair natural orbitals CC approximation, namely DLPNO-CCSD-(T) was applied to three spin crossover model complexes. The performance of this method was found to be very similar to that of the conventional CCSD­(T) but at a fraction of the cost.^31^ Lastly, a new composite method called CASPT2 + δMRCI combines CBS-extrapolated CASPT2 data with a high-level multireference configuration interaction MRCI + Q correction in a mixed basis set. This innovative approach effectively reproduces MRCI + Q/CBS energy differences for small molecular test complexes.^32^

However, all methods listed above are still computationally expensive, especially for most SCO complexes, typically consisting of ca. hundred atoms. Consequently, computationally efficient DFT methods have emerged as the preferred method for studying SCO complexes. Nonetheless, selecting the functional becomes critical in describing the exchange–correlation energy necessary to obtain accurate results. Jensen and Cirera conducted the first benchmark study on the performance of DFT methods.^33^ They analyzed the computed enthalpies of several SCO complexes containing Fe and Co atoms using six different functionals. Remarkably, the TPSSh functional accurately predicted the correct ground state for all SCO complexes. The mean absolute error (MAE) for a benchmark group of 20 mononuclear complexes was 3.7 kcal·mol^–1^, representing the best result for this type of calculation. However, this error translates to a mean deviation of 324 K in *T*_1/2_, which limits its usefulness in predicting transition temperatures.^34^ Vennelakanti et al.^22^ reported a massive study on SCO complexes. The authors examined ninety-five Fe­(II) complexes using 30 different functionals. The study found that only three functionals (modB3LYP and TPSSh, with a 10% Hartree–Fock (HF) exchange contribution, and pure M06-L) accounted for the SCO behavior of Fe complexes, i.e. yielding *T*_1/2_ between 0 and 1000 K. However, the computed *T*_1/2_ values showed limited correlation with experimentally reported values, with *r* ^2^ values of ∼0.36 for all three functionals. In a comprehensive study, Kepp^35^ analyzed 30 iron­(II) SCO systems with experimentally determined thermodynamic parameters, evaluating 12 different density functional approximations. When employing the BP86/def2-SVP/COSMO level of theory within the harmonic approximation, the computed vibrational entropies showed significant variation relative to experimental values, yielding a standard deviation of 14.6 J·mol^–1^·K^–1^ and a poor correlation coefficient of 0.52. Radoń^36^ critically analyzed the effect of using *quasi*-harmonic (QH) models to evaluate the vibrational enthalpic and entropic contributions. QH models predict a smaller change of the vibrational entropy for the SCO transition, but the observed deviations using harmonic approximation were not systematic,^35^ so that the use of simple QH models does not necessarily lead to a more accurate prediction of these entropy changes.

Since the accurate calculation of *T*_1/2_ values is extremely difficult, alternatively approaches have been proposed to rationalize how small modifications in the ligand affect the ligand field strength and consequently the *T*_1/2_ values. In their seminal study, Kershaw Cook et al.^14^ examined twenty-two [Fe^II^(bpp^X^)_2_]^2+^ complexes by substituting the para-position in the pyridine ring substituent where bpp = 2,6-di­{pyrazol-1-yl}­pyridine, observing a strong correlation between Hammett’s σ_p_ parameter of the substituent and the reported *T*_1/2_ values for the complexes. Their DFT calculations using the BP86 functional revealed that the energy gap between HS and LS states correlated with σ_p_ values, leading to the conclusion that metal–ligand π-backbonding effects are predominant. Inspired by these findings, Bondì et al.^17^ conducted an Energy Decomposition Analysis^37^ coupled with the Natural Orbitals for Chemical Valence^38,39^ (EDA-NOCV) to quantify the σ- and π-contributions to the metal–ligand bonding. Intriguingly, EDA-NOCV analysis of 16 SCO complexes from Kershaw et al.’s series of twenty-two revealed a strong correlation (*r* ^2^ = 0.82) between the observed *T*_1/2_ values and the σ-bonding descriptor Δ*E*_orb,σ_. In striking contrast, the π-bonding component Δ*E*_orb,π_ showed no meaningful correlation with *T*_1/2_ (*r* ^2^ = 0.09), pointing toward a predominant role of the σ-type metal–ligand interactions in the spin transition thermodynamics. However, the same authors performed an EDA-NOCV analysis to study the Fe–N bonds in five [Fe^II^(L^azine^)_2_(NCBH_3_)_2_] complexes, finding a noteworthy correlation between the *T*_1/2_ and the sum of both σ- and π-contributions, Δ*E*_orb,σ+π_.^15^ Rodríguez-Jiménez et al.^40^ found a promising correlation between the *T*_1/2_ values of the set of five Fe–N SCO complexes and the DFT-calculated ^15^N NMR chemical shift (δ_NA_) of the corresponding free azine-substituted compounds. To test the applicability of this descriptor, the δ_NA_ values were also predicted for a family of 13 ligands in para-substituted [Fe^II^(bpp^X^)_2_]^2+^ complexes, showing a good correlation (*r* ^2^ = 0.89) with the observed *T*_1/2_ values. This strategy was further re-evaluated by Bondì et al.^16^ by applying it to 12 [Fe^II^(pybox^X^)_2_]^2+^, seven [Fe^II^(pytacn^X^)­(CH_3_CN)_2_]^2+^ and five [Fe­(L^pytZ^)_2_(NCBH_3_)_2_] SCO complexes, finding moderately to excellent correlations with *T*_1/2_ values (*r* ^2^ = 0.69–0.96). On the other hand, Kimura and Ishida.^41^ revealed a significant correlation (*r* ^2^ = 0.73) between the partial atomic charge on the pyridine nitrogen atom obtained from the natural population analysis, and *T*_1/2_ for a set of 12 [Fe^II^(pybox^X^)_2_]^2+^ SCO complexes.

With these precedents in mind, the goal of this work is 2-fold. First, we attempt the direct calculation of the *T*_1/2_ values for the set of [Fe^II^(bpp^X^)_2_]^2+^ complexes from first-principles, with particular attention to (i) the critical evaluation of density functional performance for spin-state energetics, (ii) the quantitative assessment of temperature effects on thermodynamic parameters, and (iii) the systematic investigation of quasi-harmonic corrections for low-frequency vibrational modes in the accurate determination of *T*_1/2_ values. Second, we develop robust electronic descriptors to correlate ligand electronic properties with spin-crossover behavior, enabling more efficient screening and design of SCO materials.

### Methodology

The SCO phenomenon can be described as the thermodynamic equilibrium between two spin states as denoted in eq 1.

1 LS⇆HS

The standard Gibbs free energy difference for the spin transition can be written as^42^

2 $$\Delta G^{HS-LS}\left(T\right)=G^{HS}-G^{LS}=\Delta H^{HS-LS}\left(T\right)-T\Delta S^{HS-LS}\left(T\right)$$

where Δ*H* ^HS–LS^(*T*) and Δ*S* ^HS–LS^(*T*) are the enthalpy and entropy differences between the HS and LS states, respectively. The enthalpy change can be broken down as

3 $$\Delta H\left(T\right)={\Delta E}_{el}^{HS-LS}+\Delta H_{vib}^{HS-LS}\left(T\right)$$

where Δ*E*_el_^HS–LS^ is the electronic energy difference, directly computed from ab initio calculations, and Δ*H*_vib_^HS–LS^(*T*)­is the difference in the vibrational enthalpy correction. The entropy changes contain an electronic and vibrational contribution (and a minor rotational)

4 $$\Delta S^{HS-LS}\left(T\right)={\Delta S}_{el}^{HS-LS}+{\Delta S}_{vib}^{HS-LS}\left(T\right)$$

The former can be trivially obtained through the spin multiplicity of each spin state

5 $$S_{elec}=R\;\ln\left(2S+1\right)$$

The temperature at which Δ*G* ^HS–LS^(*T*) vanishes is known as the *T*_1/2_ and is simply given by

6 $$T_{1/2}=\frac{\Delta H^{HS-LS}\left(T_{1/2}\right)}{\Delta S^{HS-LS}\left(T_{1/2}\right)}$$

Different assumptions can be made to compute *T*_1/2_ from eq 6. First of all, although Δ*H* ^HS–LS^ and Δ*S* ^HS–LS^ exhibit some temperature dependence, this could be neglected.^43,44^ Vibrational contributions to the entropy and enthalpy for each spin state are typically obtained using the rigid-rotor-harmonic oscillator (RRHO) approximation,^45^ namely

7 $$H_{vib}\left(T\right)=\sum_{i}^{N_{vib}}\left(\frac{1}{2}h\nu_{i}+\frac{h\nu_{i}e^{-h\nu_{i}/k_{B}T}}{1-e^{-h\nu_{i}/k_{B}T}}\right)$$

and

8 $$S_{vib}\left(T\right)=\sum_{i}^{N_{vib}}\left(\frac{h\nu_{i},1}{Te^{h\nu_{i}/k_{B}T}-1}-k_{B}\;\ln\left(1-e^{-h\nu_{i}/k_{B}T}\right)\right)$$

However, it has been argued that the RRHO approximation poorly describes the contributions of low-frequency vibrational modes, which are also prone to numerical errors.^46^ To overcome this limitation, quasi-harmonic (QH) models can be used as a straightforward extension of the harmonic approximation. Two primary methods have been proposed in the literature. The simpler approach, introduced by Cramer and Truhlar,^47^ involves scaling all frequencies below a specified cutoff (usually 100 cm^–1^) up to that threshold before computing entropy within the RRHO approximation. Alternatively, Grimme replaced the contribution of low-frequency modes to the entropy below a certain cutoff by a corresponding free-rotor rotational entropy.^46^ He then used a Head-Gordon’s damping function^48^ to continuously interpolate between the free-rotor and harmonic vibrational approximations. Head-Gordon^49^ applied a similar rationale to correct for vibrational enthalpy contributions, interpolating between the harmonic approximation expression and that for a rigid rotor.

### Computational Details

All electronic structure calculations were performed using Gaussian16.^50^ Geometry optimizations were conducted separately for both spin states using the TPSSh functional,^51^ which has been found to perform reasonably well in predicting *T*_1/2_ ^22,33^ and BP86,^52,53^ which was used in the landmark study of Kershaw Cook et al.^14^ to study the [Fe^II^(bpp^X^)_2_]^2+^ complexes. The def2tzvp basis set was used on all atoms.^54^

The spin-resolved EFOs have been obtained with the APOST-3D program,^55^ using the topological fuzzy Voronoi cells (TFVC)^56^ atomic definition. The APOST-3D code is hosted and maintained on GitHub, specifically at the repository mgimferrer/APOST3D. The Grimme and Head-Gordon’s quasi-harmonic approximation was included to study the effect of low-frequency modes on the entropic and enthalpy terms, respectively, by employing the GoodVibes code developed by Luchini et al.^57^ Additionally, GoodVibes was utilized to assess the temperature dependence of the enthalpic and entropic terms to obtain *T*_1/2_ values.

## Results and Discussion

We examined a set of 24 SCO complexes with the general formula [Fe^II^(bpp^X^)_2_]^2+^, obtained and compiled by Kershaw Cook et al.,^14^ complemented by four additional compounds gathered by Halcrow et al.,^58^ Galadzhun et al.,^59^ and Attwood et al.^60^ The different para-substituted bpp^X^ ligands are shown in Figure 1. Their experimental *T*_1/2_ values in solution are reproduced in Table 1. The para substitution in the ligand has a significantly impact on the *T*_1/2_ values, covering a wide range of temperatures, from *T*_1/2_ = 158 K for X = OCH_3_ to *T*_1/2_ = 309 K for X = NO_2_. Notice that complexes with X = NH_2_ and X = NMe_2_ exhibit a HS ground-state down to 145 and 190 K, respectively.

**Figure 1:**
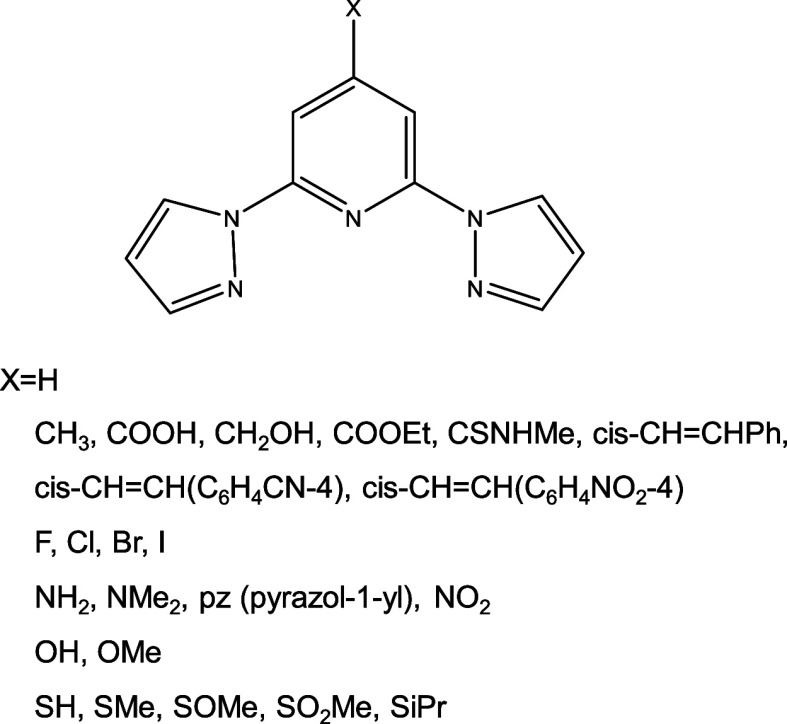
Different substituted bpp^X^ ligands studied in this work.

**Table 1:** Experimental and Calculated *T*_1/2_, Sigma Donor (σ^d^), Pi Acceptor (π^a^) and Free Ligand Resonance (*R*) Descriptors for Different Substituted (bpp^X^) Ligands in the [Fe^II^(bpp^X^)_2_]^2+^ Complexes^d^

X	exp *T*_1/2_	*T*_1/2_ ^a^	*T*_1/2_ ^b^	*T*_1/2_ ^c^	σ^d^	π^a^	*R*	refs
H	248	474	470	559	0.9422	0.4226	0.0000	^61^
CH_3_	216	453	450	535	0.9390	0.4194	0.0128	^14^
COOH	281	502	497	605	0.9478	0.4306	–0.0802	^14^
NH_2_	<145	357	356	424	0.9262	0.4084	0.1118	^14^
NMe_2_	<190	351	349	412	0.9264	0.4086	0.1236	^14^
NO_2_	309	484	479	579	0.9482	0.4316	–0.1026	^14^
pz (pyrazol-1-yl)	215	408	407	497	0.9348	0.4160	0.0162	^14^
OH	164	408	406	483	0.9302	0.4120	0.0762	^14^
OMe	158	403	402	479	0.9302	0.4120	0.0742	^14^
F	215	433	431	512	0.9336	0.4156	0.0356	^14^
Cl	226	451	448	538	0.9384	0.4196	–0.0008	^14^
Br	234	439	436	525	0.9392	0.4204	–0.0082	^14^
I	236	463	460	563	0.9398	0.4212	–0.0084	^14^
SH	246	439	437	527	0.9358	0.4172	0.0250	^62^
SCH_3_	194	421	419	505	0.9350	0.4158	0.0370	^63^
SOCH_3_	284	471	467	562	0.9420	0.4236	–0.0394	^63^
SOOCH_3_	294	500	494	597	0.9462	0.4278	–0.0830	^63^
*cis*-CH=CHPh	245	451	448	537	0.9400	0.4204	–0.0070	^64^
*cis*-CH=CH (C_6_H_4_CN-4)	259	435	433	524	0.9408	0.4214	–0.0194	^64^
*cis*-CH=CH (C_6_H_4_NO_2_-4)	261	466	462	561	0.9412	0.4216	–0.0222	^64^
CH_2_OH	229	459	456	546	0.9394	0.4196	–0.0030	^58^
SiPr	215	422	420	507	0.9350	0.4150	0.0330	^58^
COOEt	275	501	496	606	0.9466	0.4280	–0.0696	^59^
CSNHCH_3_	262	451	448	548	0.9430	0.4244	–0.0408	^60^

a Calculated using the thermodynamic data obtained at 298.15 K.

b Calculated considering the thermodynamic data dependent on the temperature.

c Calculated considering the thermodynamic data dependent on the temperature and including Grimme and Head-Gordon’s quasi-harmonic approximation.

d Values obtained at the TPSSh/def2-TZVP level of theory.

We performed full geometry optimization and harmonic frequency calculations for the whole set of SCO complexes of both LS and HS states using two different density functional approximations (BP86 and TPSSh) in combination with the def2-TZVP basis set. We considered BP86 because Kershaw Cook et al.^14^ performed geometry optimization at the BP86­(COSMO, acetone)/def2-SVP level of theory, and considered only electronic energy differences between HS and LS states. On the other hand, we also considered the TPSSh functional because it has been shown^22,33^ to perform particularly well in spin-state energetics of these SCO systems.

A comparison of the six different experimental Fe–N distances (in LS ground-state) with the optimized results for the complex [Fe^II^(bpp^H^)_2_]^2+^ is shown on Table S1. Both functionals perform extremely well, with mean average errors (MAE) of 0.011 Å and 0.009 Å for TPSSh and BP86, respectively. Additionally, both functionals account similarly for the typical geometrical changes along the spin transition. The Fe–N distances increase on average by 0.217 Å and 0.223 Å, respectively.

From the energetic point of view, both functionals predict the LS as the ground state for all selected systems (including complexes with X = NH_2_ and X = NMe_2_), as shown in Table S2. However, the magnitude of the computed Δ*E*_el_^HS–LS^ values is quite different. With the TPSSh functional, the values are in the range of 7.7–10.7 kcal·mol^–1^, whereas for BP86 they increase to a range of 22.7–25.6 kcal·mol^–1^. It is important to note that Kershaw Cook et al.^14^ reported relative Δ*E*_el_^HS–LS^ values with the BP86 functional, arbitrarily setting Δ*E*_el_^HS–LS^ = 0 for X = H. The absolute values are, however, largely overestimated with this functional.

The computed Δ*E*_el_^HS–LS^ values with both functionals show an excellent correlation (*r* ^2^ = 0.96), with a slope very close to 1, indicating a systematic deviation of ca. 15 kcal·mol^–1^ for BP86. The Δ*E*_el_^HS–LS^ values also show a significant correlation with the experimental *T*_1/2_ values (*r* ^2^ = 0.72 for BP86 and *r* ^2^ = 0.74 for TPSSh), as already noticed by Kershaw Cook et al.^14^

Including zero-point and thermal enthalpic and entropic corrections in the gas-phase at *T* = 298.15 K and with the usual RRHO approximations favor the HS state by ca. 6–7 kcal·mol^–1^. The Δ*G* ^HS–LS^ values are still too large for the BP86 functional, with values in the range of 15.6–19.0 kcal·mol^–1^ depending on the substituent. In the case of TPSSh, the Δ*G* ^HS–LS^ values are much smaller (1.1–3.9 kcal·mol^–1^), exhibiting also a somewhat tighter range. The smaller values correspond precisely to X = NH_2_ and X = NMe_2_ substituents, but still the calculations fail to predict their HS ground state nature. The correlation between the Δ*G* ^HS–LS^ values obtained with both functionals is maintained (*r* ^2^ = 0.95), even though no correlation is observed between their respective Δ*S* ^HS–LS^ values (*r* ^2^ = 0.15).

The BP86 functional consistently yields larger energy differences than the TPSSh functional. This difference is particularly noticeable when calculating the transition temperature for each system. When using the BP86 functional, too high *T*_1/2_ values are obtained (in all cases above 1000 K as shown in Table S3), using eq 6 and Δ*H* ^HS–LS^ and Δ*S* ^HS–LS^ values computed at *T* = 298.15 K and the RRHO approximations. Using the TPSSh functional yields *T*_1/2_ values ranging between 351 and 502 K, much closer to the experimental measurements but still with MAE over 212 K. Despite these large errors, there is a moderate correlation between the computed and observed transition temperature, with *r* ^2^ = 0.72 and a slope of 1.10 for the TPSSh functional, as shown in Figure 2a. The correlation for the BP86 functional is significantly worse (see Figure S6a), so from now on we will focus solely on the TPSSh results.

**Figure 2:**
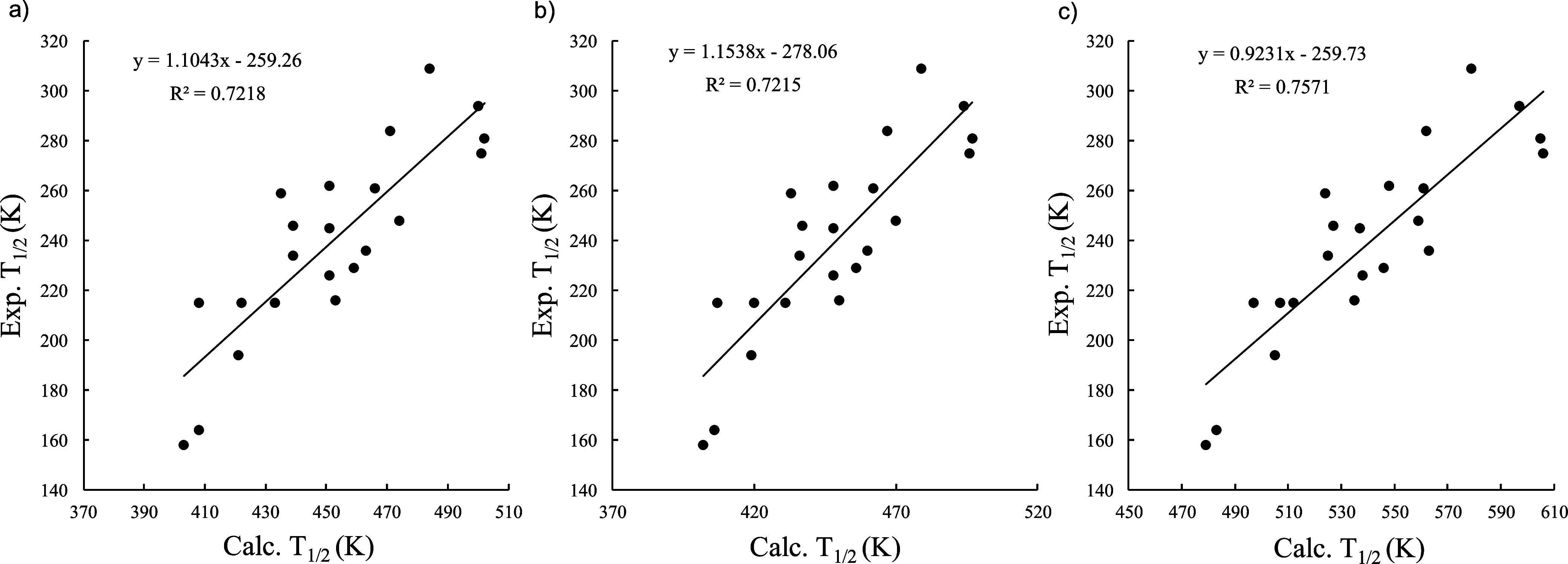
Plots of experimental *T*_1/2_ vs *T*_1/2_ predicted by (a) using the thermodynamic data obtained at 298.15 K, (b) considering the thermodynamic data dependent on the temperature and (c) considering the thermodynamic data dependent on the temperature and including Grimme and Head-Gordon’s quasi-harmonic approximation. Values obtained at the TPSSh/def2-TZVP level of theory.

As previously noted, both Δ*H* ^HS–LS^ and Δ*S* ^HS–LS^ values exhibit some temperature dependence, which is often ignored when computing the *T*_1/2_ values. We have examined this effect by recomputing these values in a range of 250–500 K using GoodVibes program and numerically finding the temperature at which Δ*G* ^HS–LS^ = 0. The results obtained for the TPSSh functional are also collected on Table 1, and the plot against experimental values in Figure 2b. The computed *T*_1/2_ are systematically smaller considering the temperature variation, but the effect is only minor (ca. 1–5 K), in line with the findings of Gütlich et al.^43,44^ The correlation with the experimental data is barely affected, too.

In addition to the temperature dependence of the enthalpic and entropic terms, we have also examined the effect of using the QH models of Grimme and Head-Gordon for the low-frequency vibrational contributions, using the conventional 100 cm^–1^ cutoff value for the harmonic frequencies. As shown in Table 1, the calculated *T*_1/2_ values are systematically higher by ca. 90 K when using the QH models, thus further deviating from the experimental reference. The increase in *T*_1/2_ is mainly due to a decrease of the Δ*S* ^HS–LS^ values. This is because the HS state usually exhibits a larger number of low-frequency normal modes, for which the QH entropic contribution is smaller than that obtained with the RROH approximation. Consequently, the MAE for the predicted *T*_1/2_ values further increase to 301.3 K. However, the *r* ^2^ of the correlation slightly increases to 0.76 (see Figure 2c).

The results indicate that the combination of the TPSSh functional and the def2-TZVP basis set provides a reliable approach for the qualitative modeling of spin-crossover behavior. However, direct computation of the transition temperature from the calculated Δ*H* ^HS–LS^ and Δ*S* ^HS–LS^ differences still leads to overwhelming deviations from experimental values.

A meaningful correlation is observed between the experimental *T*_1/2_ values and the electronic energy differences Δ*E*_el_^HS–LS^ computed with either the BP86 or TPSSh functional. This suggests the presence of a systematic error in the spin-state energetics. To test this hypothesis, we applied a constant shift to the computed Δ*H* ^HS–LS^ values, determined by setting the mean signed deviation between experimental and calculated *T*_1/2_ values to zero. The adjusted values were then used to estimate the transition temperatures. For the TPSSh functional, the required shift was −4.1 kcal·mol^–1^, resulting in a MAE of 16.8 K and a maximum deviation of 38 K. In the case of BP86, a much larger shift of −18.9 kcal·mol^–1^ was needed, but the resulting MAE (17.0 K) and maximum deviation (39 K) were nearly identical to those obtained with TPSSh. This indicates that, after correction, both functionals yield transition temperature predictions of similar accuracy.

In order to reach a quantitative prediction of the *T*_1/2_ values, a better strategy is to find suitable descriptors able to capture the delicate electronic effects induced by the ligand substitution. In this sense, we have recently shown how the so-called effective fragment orbitals (EFOs)^65,66^ and their occupations can be used to describe the electronic substituent effect and to quantify donor/acceptor interaction between metal and ligands.^67,68^ The spin-resolved EFOs can be understood as the fragment domain natural spinorbitals, with fractional occupations between 0 and 1. They are easily obtained for each fragment or atom individually considered (typically the metal centers and the ligands), using essentially any underlying atom-in-molecule definition. The EFOs of a ligand within a molecular complex can be put into correspondence with the molecular orbitals of the free ligand, but exhibiting fractional occupations as a result of the interaction of the ligand with the metal and other ligands.

Let us illustrate how the EFOs and their occupations can be used to construct proper quantitative models for the *T*_1/2_ values in SCO complexes. The shape and occupation of the relevant spin-resolved EFOs for the bpp^H^ ligand within the [Fe^II^(bpp^H^)_2_]^2+^ complex in their LS ground state are depicted in Figure 3. Since the LS state is described by a closed-shell single determinant, the alpha and beta EFOs and their occupations are exactly the same. Here we will focus solely on the alpha part. One can identify six EFOs involved in the metal–ligand bonding, which can be categorized into σ-type and π-type interactions. The bpp^H^ ligand exhibits three EFOs with σ character directed toward the Fe center, with net occupations of 0.893, 0.835, and 0.737, respectively, in line with its tridentate character. These EFOs do not directly correspond to the three individual σ bonds but to appropriate lineal combinations of them, as shown in Figure 3. The σ donation from the ligand to the metal results in a decrease of the occupation of the EFOs from the ideal 1 down to the occupations mentioned above. The difference from all EFOs with the appropriate σ symmetry accounts for the total amount of σ donation, σ^d^, from the bpp^H^ ligand to the metal

9 $$\sigma^{d}=2\sum_{k}\left({1-\lambda }_{k}^{\sigma }\right)$$

where λ_*k*_^σ^ represent the occupation of the EFOs characterizing the σ-type metal–ligand interaction, and the factor two originates from the alpha and beta contributions. Similarly, Figure 3 also shows three additional EFOs that describe π-type interactions between the metal and the ligand, with very small but not negligible occupations of ca. 0.05–0.1. These EFOs can be put into correspondence with virtual MOs of the free bpp^H^ ligand, and are thus empty in the ideal case. Thus, these occupations account for the amount of π backdonation from the metal to the ligand, completing the classical Dewar-Chat-Duncanson model.^69^ The extent of π backdonation can be expressed from the sum of the occupations of the EFOs with appropriate π character as

10 $$\pi^{a}=2\sum_{k}\lambda_{k}^{\pi }$$

Focusing on the σ-donor properties of the bpp^X^ ligands, the highest value of the σ^d^ descriptor is found for X = NO_2_, followed by some C-based substituents such as X = COOH, COOEt, or CSNHCH_3_. On the other hand, the weakest σ-donor ligands are the ones with substituents X = OH, OMe, NMe_2,_ and NH_2_. It is important to note that the X = NO_2_ and X = NH_2_ substituted ligands are at opposite ends of the σ-donor scale, indicating that the substituent effect is not only determined by the contact atom but by its chemical environment. Remarkably, the largest experimental *T*_1/2_ value among the set corresponds to the X = NO_2_ substituted ligand, while the X = NH_2_ substituted ligand exhibits a HS state over 145 K. Therefore, the stronger the σ-donation from the ligand, the higher the experimental *T*_1/2_. This trend is corroborated by the excellent correlation depicted in Figure 4.

**Figure 3:**
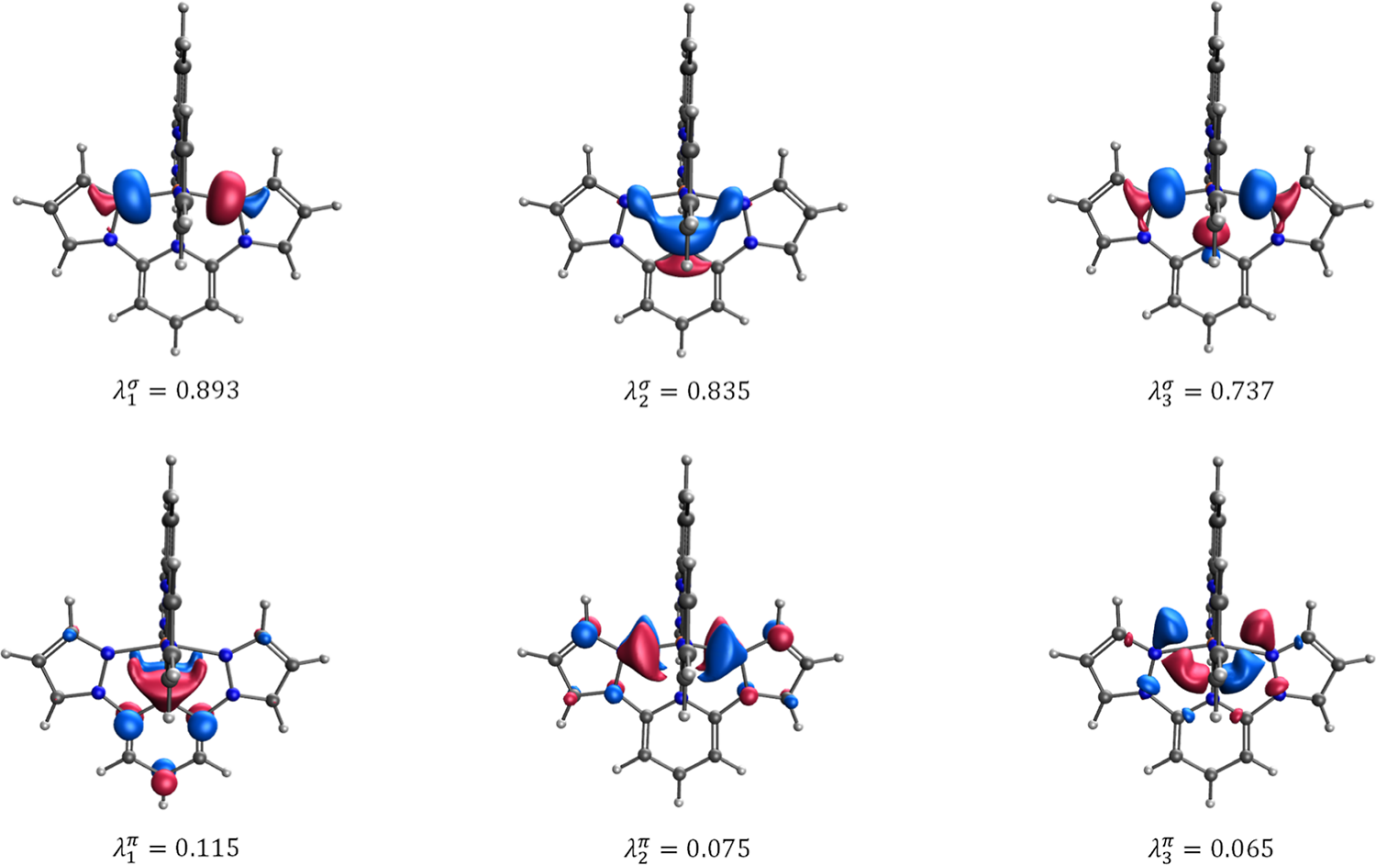
Shapes and occupancies of the EFOs characterizing the bpp^H^ ligand → metal σ-donation and metal → bpp^H^ ligand π-backdonation in the [Fe^II^(bpp^H^)_2_]^2+^ complex.

**Figure 4:**
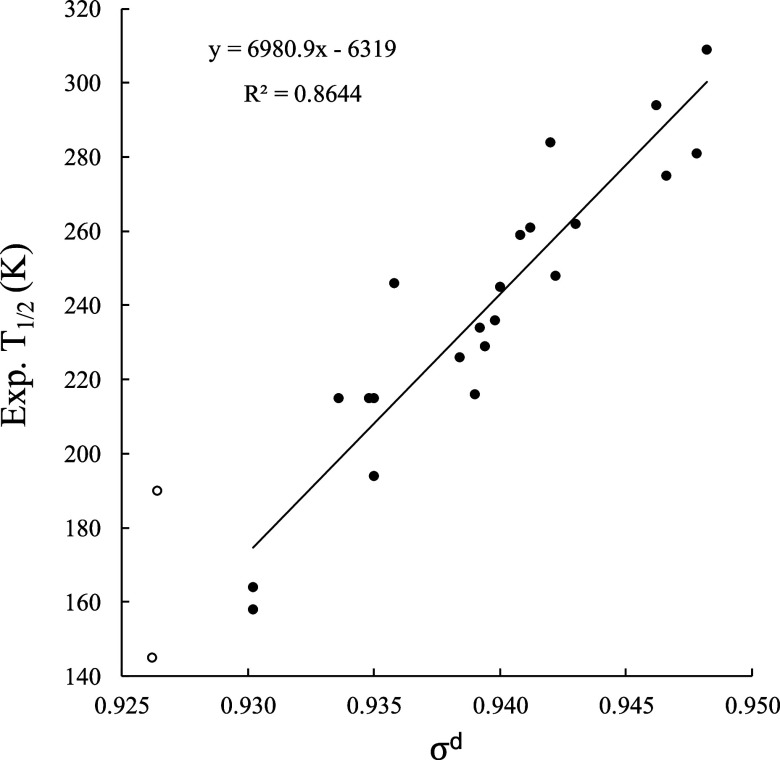
Linear relationship between the experimental *T*_1/2_ values and the σ^d^ descriptor for the set of [Fe^II^(bpp^X^)_2_]^2+^ complexes obtained at the TPSSh/def2-TZVP level of theory. Empty circles (not used for the correlation) correspond to the upper limit values for X = NH_2_ and X = NMe_2_ (see text).

We have determined the σ^d^ and π^a^ descriptors for the set of LS [Fe^II^(bpp^X^)_2_]^2+^ complexes at the TPSSh/def2-TZVP level of theory. The shape of the key EFOs is essentially unchanged with the ligand substitution, but the respective occupations are slightly modified according to their electron donating or electron withdrawing character. The descriptors obtained using eqs 9 and 10 are also collected in Table 1.

These findings align with the work of Bondì et al.,^17^ which established a similar correlation (*r* ^2^ = 0.82) between Δ*E*_orb,σ_ (from EDA-NOCV analysis) and *T*_1/2_ for a subset of 16 ligands. Our extended study of 24 ligands shows improved correlation (*r* ^2^ = 0.86), with a MAE of 11.8 K and a maximum deviation of 32.3 K for X = SH. This robust correlation suggests that variations in σ-donation upon ligand substitution would primarily govern the observed shifts in transition temperatures.

However, the amount of π backdonation also exhibits very good and positive correlation with the experimental *T*_1/2_ values, as shown in Figure 5. We obtain *r* ^2^ = 0.85, with a MAE of 11.9 K and a maximum deviation of 29.8 K, again for X = SH. This indicates that ligands with better π-acceptor properties, incorporating electron withdrawing groups (EWGs), enhance the ligand field, favoring the LS state and increasing the *T*_1/2_ values. This is more in line with Kershaw Cook et al.’s original proposal,^14^ which attributed the *T*_1/2_ trends to dominant π-bonding effects based on correlations with Hammett σ_p_^+^ parameters. It is, however, in sharp contrast with the analysis of Bondì et al.,^17^ who found a poor correlation (*r* ^2^ = 0.09) between *T*_1/2_ and the Δ*E*_orb,π_ values for the π-bonding channels.

**Figure 5:**
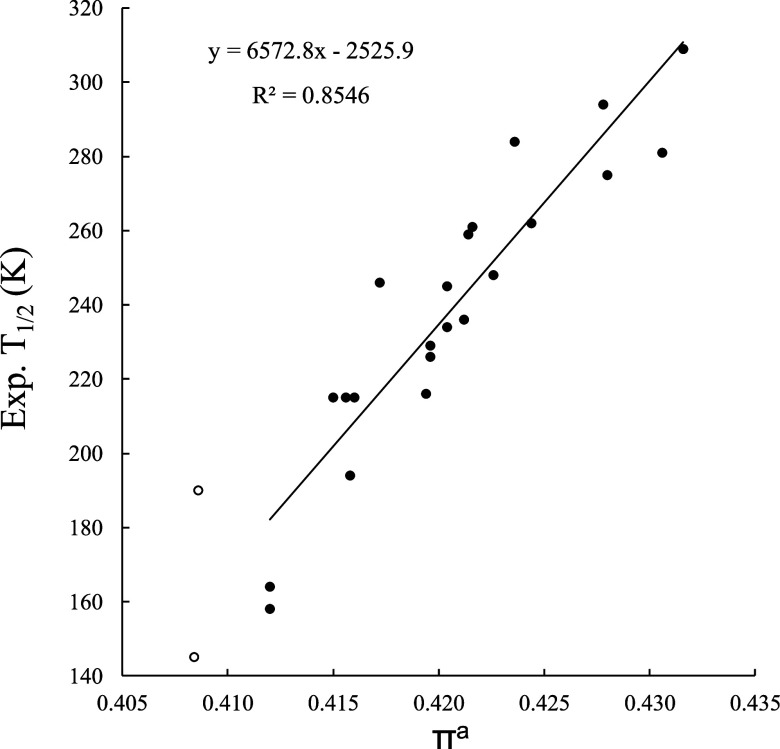
Linear relationship between the experimentally *T*_1/2_ and the π^a^ descriptor for the set of [Fe^II^(bpp^X^)_2_]^2+^ complexes obtained at the TPSSh/def2-TZVP level of theory. Empty circles (not used for the correlation) correspond to the upper limit values for X = NH_2_ and X = NMe_2_ (see text).

While the literature primarily discusses the competition between σ and π interactions and which is more influential in predicting *T*_1/2_, this work demonstrates that both effects are equally important when they are properly accounted for. We have also calculated the σ^d^ and π^a^ descriptors using BP86 functional. Figures S7–S8 and Table S3 of the Supporting Information clearly show that the nature of the functional has minimal impact. Although the σ^d^ and π^a^ indexes obtained with the BP86 functional are systematically somewhat larger, the same correlations with *T*_1/2_ are obtained, with similar *r* ^2^ values.

Previous studies have employed density- or energy-based descriptors to characterize SCO complexes. While our current work follows this established approach, it is important to note that using the EFOs offers a distinct advantage over EDA-NOCV: it eliminates the need for additional reference calculations on free ligands while still enabling quantitative analysis of metal–ligand bonding effects.

A more efficient strategy would involve deriving descriptors directly from the free bpp^X^ ligands prior to complex formation. Such an approach would significantly streamline the computational process, facilitating rapid screening of potential ligands. The underlying premise is that ligand modifications induce subtle electronic perturbations that propagate through to the metal–ligand interactions, ultimately affecting the SCO properties. This forward-looking methodology could provide a powerful tool for predictive ligand design in SCO systems. In this case, σ^d^ and π^a^ descriptors cannot be used, the corresponding σ- and π-type MOs are either occupied or virtual. Instead, one can make use of the effective atomic orbitals (eff-AOs) of the individual atoms of the ligand. We have recently shown that^70^ the resonant effect caused by a group X into the aromatic ring of monosubstituted benzene derivatives can be captured from the occupations of the 2p_*z*_-type eff-AOs of all carbon atoms of the ring except for the ipso carbon. In particular, the difference in sum of the alpha and beta occupations of the 2p_*z*_ eff-AOs with respect to X = H serves as a descriptor for resonant effect. We extend this idea in this particular example and construct a descriptor from the occupations of the 2p_*z*_-type eff-AOs of all carbon and nitrogen atoms of the bpp^X^ ligand core, except for the carbon atom in the ipso position with respect to substituent X. The shape and occupations of these eff-AOs for the bpp^H^ ligand are overlaid in Figure 6.

**Figure 6:**
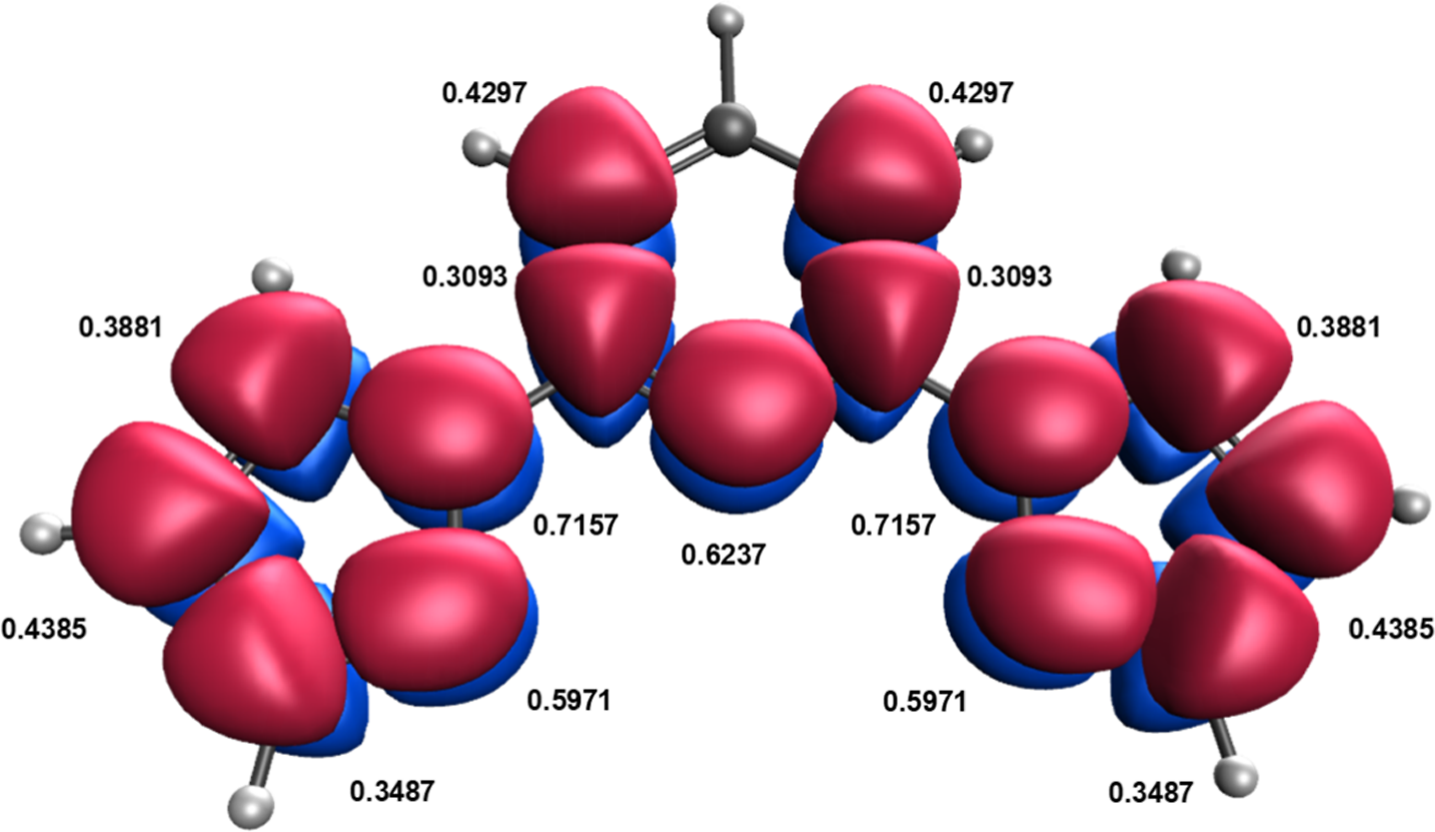
2p_*z*_ EFO shapes and occupancies of the carbon and nitrogen atoms in the backbone of the bpp^H^ ligand.

The occupations of the 2p_*z*_-type eff-AOs for the whole set of SCO complexes is gathered in Tables S4 and S5 of the Supporting Information. The positions mostly affected by the ligand substitutions are those in ortho and para of the central the pyridine ring. Their individual variations show significant correlation with the *T*_1/2_ values, with *r* ^2^ of ca. 0.7. However, it is the sum over all atoms (except the ipso carbon) relative to the value for X = H

11 $$R=2\sum_{i\in ring^{\prime }}\lambda_{i}^{2pz,X}-\lambda_{i}^{2pz,H}$$

which serves as an excellent descriptor for the ligand substitution effect, accounting for the shift in the overall π density on the ring. Thus, we find *R* > 0 for EDG substituents, and *R* < 0 for EWG substituents. The correlation of this descriptor and the experimental *T*_1/2_ values, is shown in Figure 7. A very similar correlation is obtained with the computed Δ*E*_el_^HS–LS^ values for each of the functionals.

**Figure 7:**
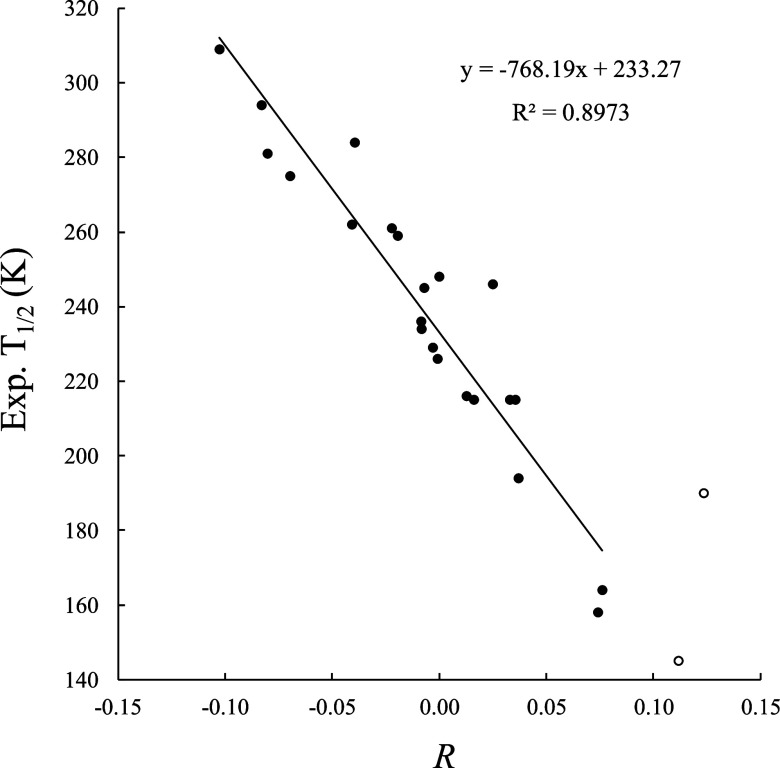
Linear relationship between the experimentally *T*_1/2_ and the *R* descriptor in the different substituted free bpp^X^ ligands for the set of [Fe^II^(bpp^X^)_2_]^2+^ complexes obtained at the TPSSh/def2-TZVP level of theory. Empty circles (not used for the correlation) correspond to the upper limit values for X = NH_2_ and X = NMe_2_ (see text).

The plot shows that positive values of *R* (i.e., for EDGs) leads to lower *T*_1/2_. Thus, the descriptors *R* and π^a^ show opposite trends (see Figure S9 of the Supporting Information). The rationale is that π-electron rich ligands will be worst π-acceptors upon interaction with the metal. We obtain an improved *r* ^2^ = 0.90 for the fit, with MAE of 10.1 K and a maximum deviation of 31.9 K. The outlier is again X = SH. Discarding X = SH from the fit leads to a further improved *r* ^2^ = 0.93 and a MAE of 8.7 K. The predicted *T*_1/2_ values for X = NH_2_ and X = NMe_2_ substituted ligands are 143.0 and 133.6 K, respectively, the lowest among the whole set. Also, the same analysis performed using the BP86 functional leads virtually to the same results (see Figure S10), underscoring the robustness of our approach.

To further validate our new descriptor, we applied it to another series of SCO complexes reported by Kimura and Ishida.^41^ These compounds, with the general formula [Fe^II^(pybox^X^)_2_]^2+^, feature 2,6-bis­(oxazolin-2-yl)­pyridine ligands substituted at the 4-position of the pyridine ring (denoted as X, see Figure 8). This family exhibits *T*_1/2_ values spanning from 170 to 310 K, providing an excellent test case for further assessing the predictive capability of our approach.

**Figure 8:**
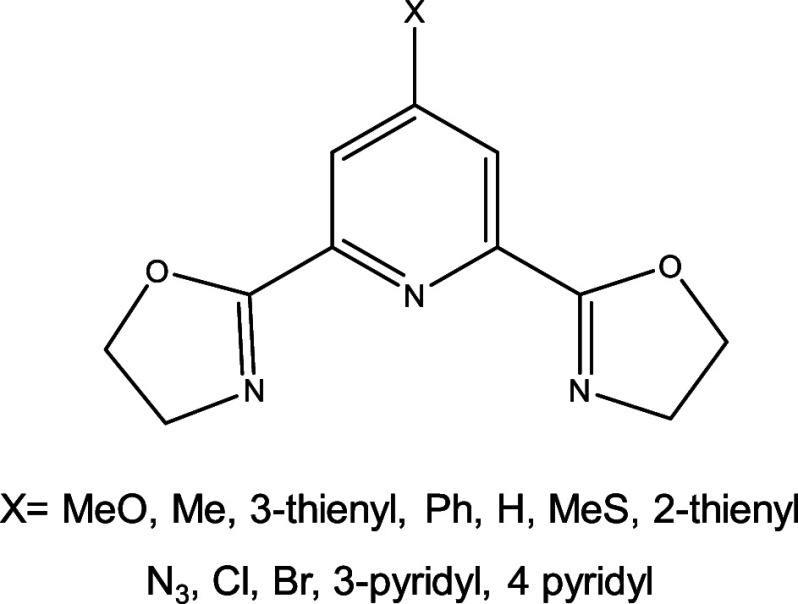
Different substituted pybox^X^ ligands studied in this work.

Kimura and Ishida^41^ found a significant correlation between the *T*_1/2_ values and the partial charge on the pyridinic nitrogen atom computed with natural population analysis (*r* ^2^ = 0.73). A somewhat less satisfactory correlation was also derived from the chemical shift of this nitrogen nucleus (*r* ^2^ = 0.69). In our study, we extended this analysis by applying the same methodology used for the [Fe^II^(bpp^X^)_2_]^2+^ complexes. Specifically, we performed full geometry optimizations of the LS ground state at the TPSSh/def2-TZVP level of theory and employed the ligand’s EFOs and eff-AOs to derive the σ^d^ and π^a^ descriptors for the complexes, along with the R descriptor of the ligands. The resulting correlation with experimental *T*_1/2_ values is presented in Figure 9.

**Figure 9:**
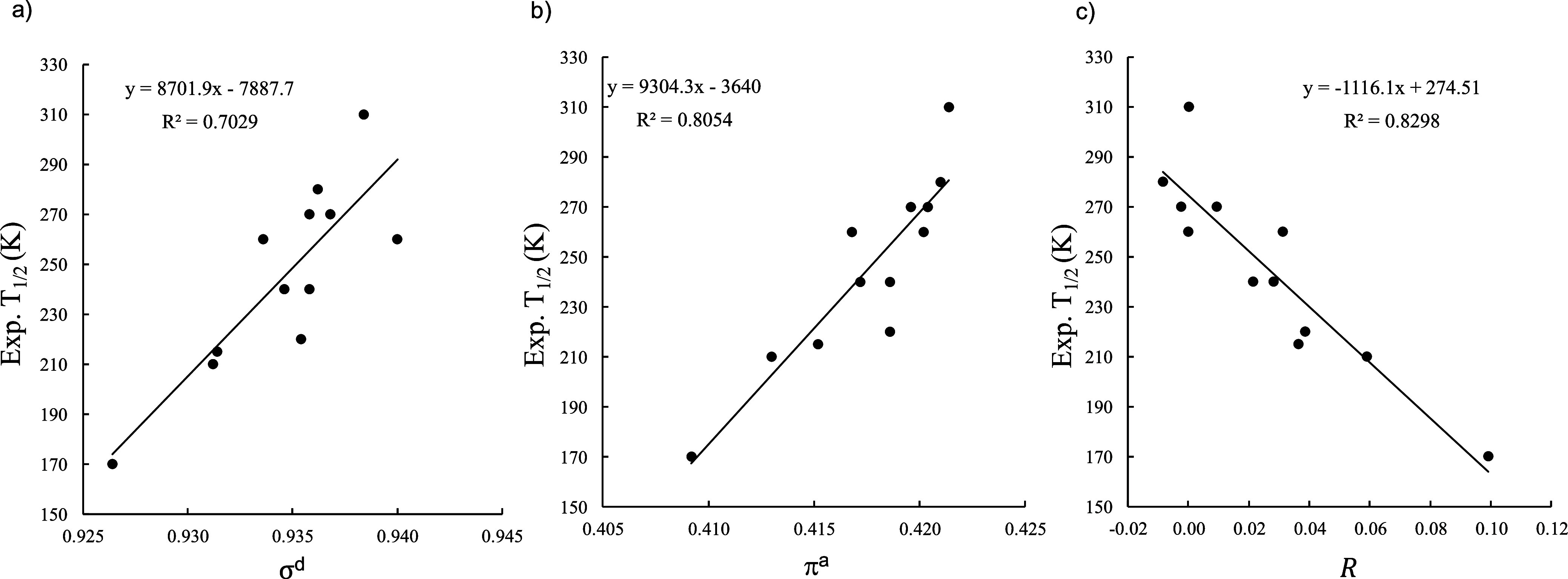
Linear relationship between the experimental *T*_1/2_ values for the set of [Fe^II^(pybox^X^)_2_]^2+^ complexes and (a) σ^d^ descriptor for σ-donation, (b) π^a^ descriptor for π acceptor character, and (c) the *R* descriptor for the free ligands, obtained at TPSSh/def2-TZVP level of theory.

The replacement of pyrazole rings with oxazole moieties would be expected to reduce π-electron delocalization across the ligand framework. Nevertheless, the computed descriptor magnitudes remain comparable to those obtained for the [Fe^II^(bpp^X^)_2_]^2+^ complexes. For this family of compounds, the correlation with *T*_1/2_ values is somewhat weaker, particularly for the σ^d^ descriptor (*r* ^2^ = 0.70). Significantly, the ligand’s *R* descriptor yields again the best correlation (*r* ^2^ = 0.83), which represents a marked improvement over the optimal correlation reported by Kimura and Ishida.^41^ In this family of compounds, the substituent X = 4-pyridyl is a clear outlier, exhibiting a *T*_1/2_ much higher than expected.

## Conclusions

We have provided a thorough computational analysis of spin-crossover behavior in [Fe^II^(bpp^X^)_2_]^2+^ complexes. By means of DFT calculations, we show that the hybrid TPSSh functional, combined with the def2-TZVP basis set, provides reasonably accurate predictions of spin-state energetics compared to other functionals, though deviations in the transition temperature (*T*_1/2_) estimates are still too large. Incorporating temperature-dependent and quasi-harmonic corrections for low-frequency vibrational contributions to enthalpic and entropic terms did not yield substantial improvements.

To enhance the accuracy of *T*_1/2_ predictions, we explore for the first time electronic descriptors based upon the occupations of effective fragment orbitals (EFOs). We show that the σ-donation and π-acceptor features of the ligands within the SCO complexes can be quantified from the occupations of appropriate σ- and π-type frontier EFOs of the ligand. Furthermore, we also introduce a simple descriptor (*R*) that accounts for the resonance effect of ligand substitution, and only requires the evaluation of the effective atomic orbitals (eff-AOs) of the isolated ligand. The observed correlations are excellent, but not perfectly transferrable between different families of SCO complexes.

Our analysis shows that incorporating of electron donating groups (EDGs) leads to more π-electron rich ligands, with a concomitant decrease in their σ-donor and π-acceptor character, resulting in lower *T*_1/2_ values. Our new approach offers a computationally efficient strategy for fine-tuning spin-state properties in transition metal complexes, facilitating the rational design of SCO systems.

## Supplementary Material





## Notes

Additional tables and figures, and occupations of all eff-AOs (PDF)
XYZ coordinates of all structures considered in this word (XYZ)
