# Supplementary material for: Rationalizing Spin-Crossover Properties of Substituted Fe (II) Complexes
Source: Inorg Chem. 2025 Jul 23;64(30):15437–47. doi: 10.1021/acs.inorgchem.5c01523 (PMC12326360; doi:10.1021/acs.inorgchem.5c01523)
Supplement: Supplementary file 1 [file ic5c01523_si_001.pdf]

Supporting Information for

Rationalizing spin-crossover properties of substituted Fe (II) complexes

Gerard Comas-Vilà,[1] and Pedro Salvador[1,]*

[1] Institut de Química Computacional i Catàlisi i Departament de Química, Universitat de Girona, c/M Aurelia Capmany 69, 17003, Girona, Catalonia, Spain.

Table S1: Calculated Fe-N bond lengths (Å) for $[Fe^{II}(bpp^{H})_2]^{2+}$ with TPSSh and BP86 functionals compared to experiment values.

Method	$[Fe^{II}(bpp^{H})_2]^{2+}$ LS			$[Fe^{II}(bpp^{H})_2]^{2+}$ HS	
	Experimental	TPSSh	BP86	TPSSh	BP86
Fe-N1 {pyridine}	1.902	1.905	1.897	2.142	2.135
Fe-N2 {pyrazole}	1.975	1.984	1.972	2.191	2.187
Fe-N3 {pyrazole}	1.972	1.984	1.972	2.191	2.187
Fe-N4 {pyridine}	1.903	1.905	1.897	2.142	2.135
Fe-N5 {pyrazole}	1.991	1.984	1.972	2.191	2.187
Fe-N6 {pyrazole}	1.951	1.984	1.972	2.191	2.187

Table S2: Computed Electronic, Enthalpy, Entropy, and Gibbs Energy Differences (High Spin – Low Spin) for the 24 systems examined in this work. Electronic, enthalpies and Gibbs energies in kcal·mol^{-1} units. Entropies in kcal·mol^{-1}·K^{-1}.

	BP86				TPSSh			
X	ΔE_e^{HS-LS}	ΔH^{HS-LS}	ΔS^{HS-LS}	ΔG^{HS-LS}	ΔE_e^{HS-LS}	ΔH^{HS-LS}	ΔS^{HS-LS}	ΔG^{HS-LS}
H	25.03	24.20	0.01935	18.43	10.31	9.38	0.01979	3.48
CH_3	24.47	23.65	0.01917	17.94	9.87	8.95	0.01974	3.06
COOH	25.55	24.67	0.01912	18.97	10.70	9.75	0.01942	3.96
NH_2	22.71	22.01	0.02005	16.04	7.79	7.10	0.01989	1.17
NMe_2	22.73	22.01	0.02161	15.56	7.80	7.07	0.02017	1.12
NO_2	25.17	24.27	0.01898	18.61	10.29	9.33	0.01927	3.58
pz (pyrazol-1-yl)	23.74	22.96	0.01933	17.19	9.26	7.95	0.01950	2.83
OH	23.22	22.48	0.01958	16.65	8.55	7.80	0.01913	2.10
OMe	23.32	22.58	0.01965	16.72	8.57	7.81	0.01936	2.04
F	23.64	22.87	0.01931	17.11	9.10	8.29	0.01911	2.59
Cl	24.10	23.29	0.01921	17.57	9.56	8.71	0.01933	2.95
Br	24.20	23.39	0.01925	17.65	9.65	8.72	0.01989	2.80
I	24.34	23.52	0.01927	17.77	9.84	8.97	0.01936	3.20
SH	23.69	22.91	0.01933	17.14	9.13	8.32	0.01894	2.67
SCH_3	23.66	22.87	0.01951	17.06	9.02	8.20	0.01949	2.39
$SOCH_3$	24.69	23.88	0.01892	18.24	10.06	9.15	0.01943	3.36
$SOOCH_3$	25.22	24.35	0.01914	18.64	10.42	9.48	0.01898	3.82
cis-CH=CHPh	24.21	23.42	0.01853	17.89	9.80	8.90	0.01971	3.02
cis-CH=CH (C_6H_4CN-4)	24.34	23.53	0.01900	17.86	9.83	8.90	0.02046	2.80
cis-CH=CH ($C_6H_4NO_2$-4)	24.41	23.60	0.01917	17.88	9.96	9.12	0.01959	3.28
CH_2OH	24.61	23.79	0.01905	18.11	10.01	9.08	0.01977	3.19
SiPr	23.61	22.84	0.01917	17.12	8.94	8.15	0.01932	2.39
COOEt	25.53	24.66	0.01893	19.02	10.73	9.78	0.01951	3.96
$CSNHCH_3$	24.59	23.75	0.02026	17.71	10.27	9.33	0.02070	3.16

Table S3: Experimental and calculated $T_{1/2}$, sigma-donor (σ^d), pi acceptor (π^a) and free ligand resonance (*R*) descriptors for different substituted (bpp^X) ligands in the $[Fe^{II}(bpp^X)_2]^{2+}$ complexes. All values obtained at the BP86/def2-TZVP level of theory.

X	Exp $T_{1/2}$	$T_{1/2}$[(a)]	$T_{1/2}$[(b)]	$T_{1/2}$[(c)]	σ^d	π^a	*R*
H	248	1250	1208	1691	1.0706	0.5100	0.0000
CH_3	216	1234	1192	1692	1.0664	0.5058	0.0144
COOH	281	1290	1243	1776	1.0790	0.5190	-0.0760
NH_2	< 145	1098	1070	1568	1.0542	0.4924	0.1162
NMe_2	< 190	1018	994	1471	1.0542	0.4926	0.1268
NO_2	309	1279	1231	1746	1.0802	0.5212	-0.0894
pz (pyrazol-1-yl)	215	1188	1150	1648	1.0626	0.5022	0.0188
OH	164	1148	1116	1593	1.0584	0.4966	0.0816
OMe	158	1149	1116	1592	1.0582	0.4964	0.0798
F	215	1184	1148	1625	1.0624	0.5010	0.0446
Cl	226	1213	1173	1669	1.0660	0.5060	0.0088
Br	234	1215	1175	1691	1.0666	0.5068	0.0004
I	236	1220	1180	1709	1.0672	0.5076	-0.0016
SH	246	1185	1149	1654	1.0628	0.5024	0.0330
SCH_3	194	1172	1136	1629	1.0618	0.5014	0.0460
$SOCH_3$	284	1262	1218	1731	1.0700	0.5098	-0.0332
$SOOCH_3$	294	1273	1227	1749	1.0748	0.5158	-0.0790
cis-CH=CHPh	245	1264	1221	1741	1.0696	0.5068	-0.0050
cis-CH=CH (C_6H_4CN-4)	259	1238	1197	1711	1.0694	0.5094	-0.0162
cis-CH=CH ($C_6H_4NO_2$-4)	261	1231	1190	1706	1.0714	0.5092	-0.0200
CH_2OH	229	1249	1206	1708	1.0668	0.5060	-0.0014
SiPr	215	1191	1154	1667	1.0616	0.5004	0.0432
COOEt	275	1303	1255	1792	1.0772	0.5160	-0.0666

$CSNHCH_3$	262	1172	1135	1650	1.0706	0.5114	-0.0348

(a) calculated using the thermodynamic data obtained at 298.15 K.

(b) calculated considering the thermodynamic data dependent on the temperature.

(c) calculated considering the thermodynamic data dependent on the temperature and including Grimme and Head-Gordon's quasi-harmonic approximation.

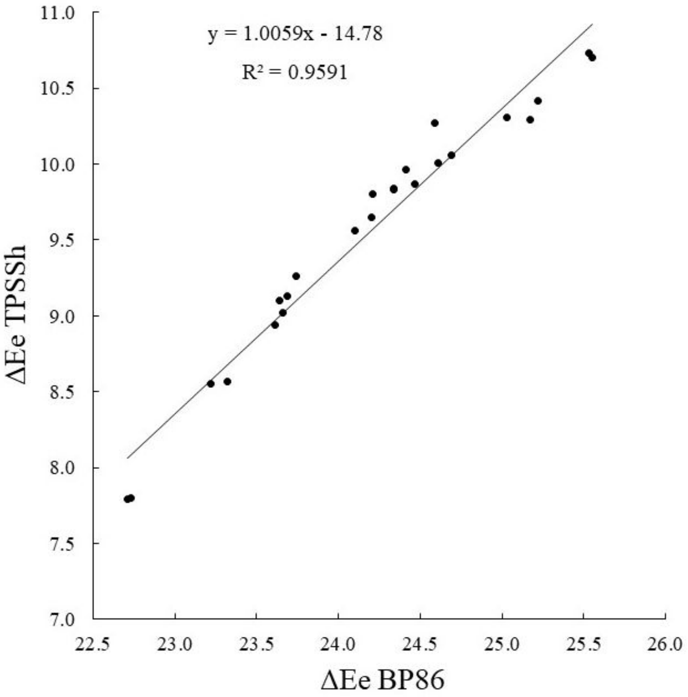

Figure S1: Effect of the functional on the electronic energy difference between high-spin and low-spin configurations for the set of $[Fe^{II}(bpp^{X})_2]^{2+}$ SCO complexes.

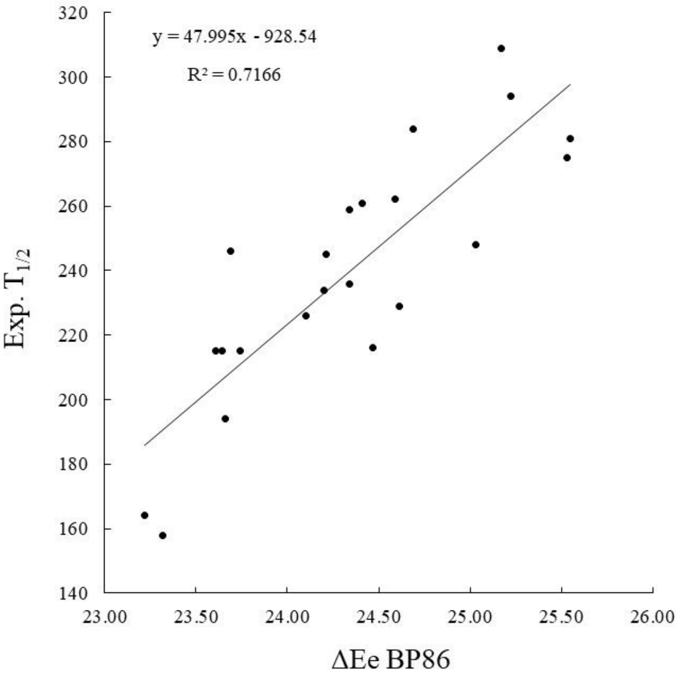

Figure S2: Experimental $T_{1/2}$ *vs* the computed ΔEe (HS-LS) using the BP86 functional for the set of $[Fe^{II}(bpp^{X})_2]^{2+}$ SCO complexes.

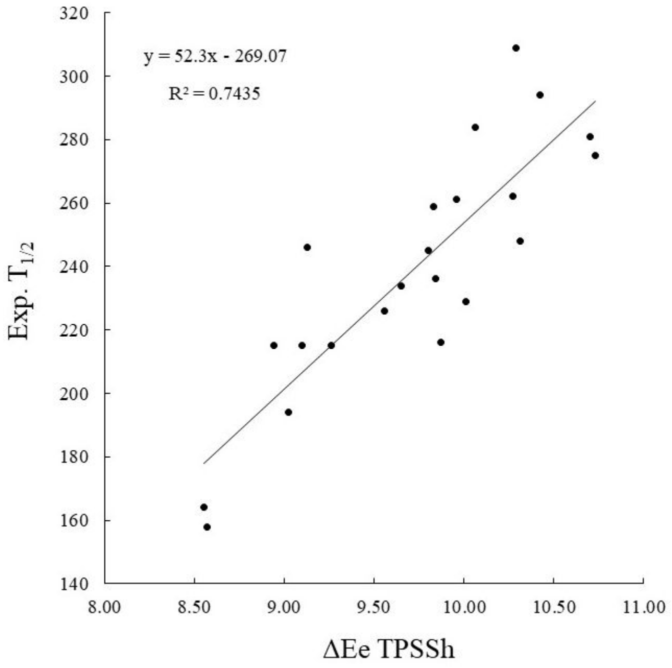

Figure S3: Experimental $T_{1/2}$ *vs* the computed ΔEe (HS-LS) using the TPSSh functional for the set of $[Fe^{II}(bpp^{X})_2]^{2+}$ SCO complexes.

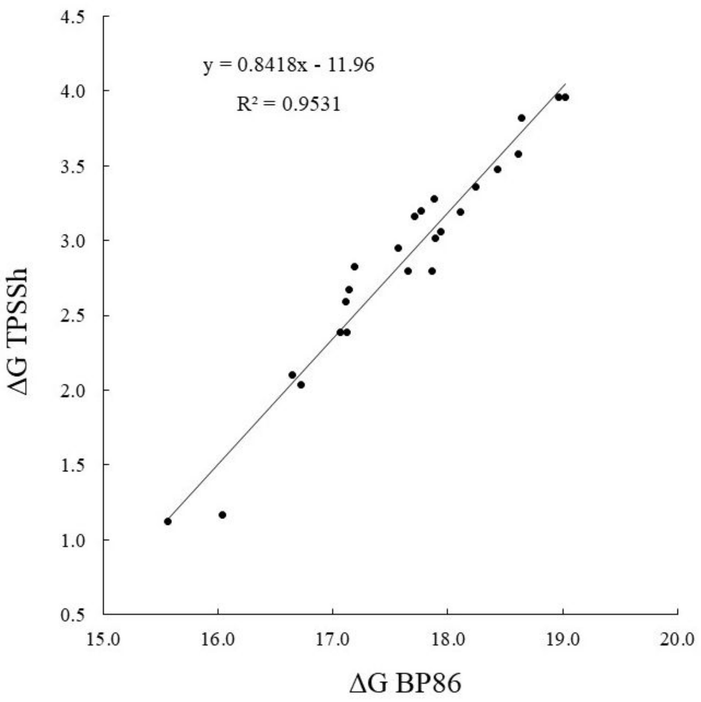

Figure S4: Effect of the functional on the Gibbs Free energy difference between high-spin and low-spin configurations for the set of $[Fe^{II}(bpp^{X})_2]^{2+}$ SCO complexes.

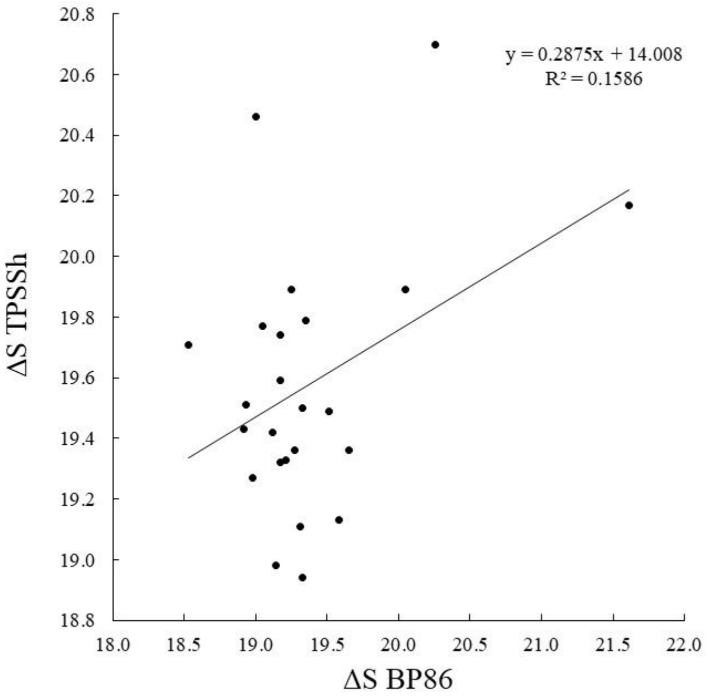

Figure S5: Effect of the functional on the entropy energy difference between high-spin and low-spin configurations for the set of $[Fe^{II}(bpp^X)_2]^{2+}$ SCO complexes. Entropies in cal·mol^{-1} K^{-1}.

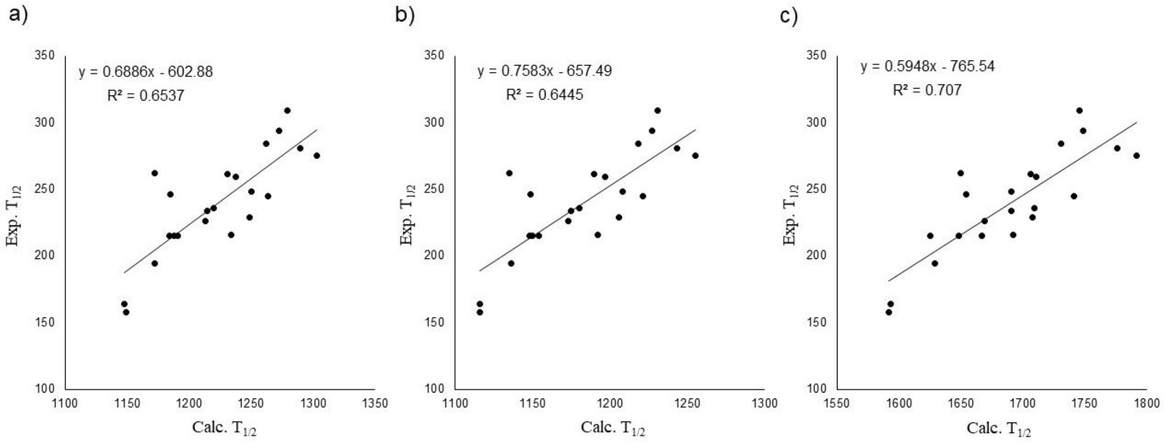

Figure S6: Plots of experimental $T_{1/2}$ *vs* $T_{1/2}$ predicted by (a) using the thermodynamic data obtained at 298.15 K, (b) considering the thermodynamic data dependent on the temperature and (c) considering the thermodynamic data dependent on the temperature and including Grimme and Head-Gordon's quasi-harmonic approximation. Values obtained at the BP86/def2-TZVP level of theory.

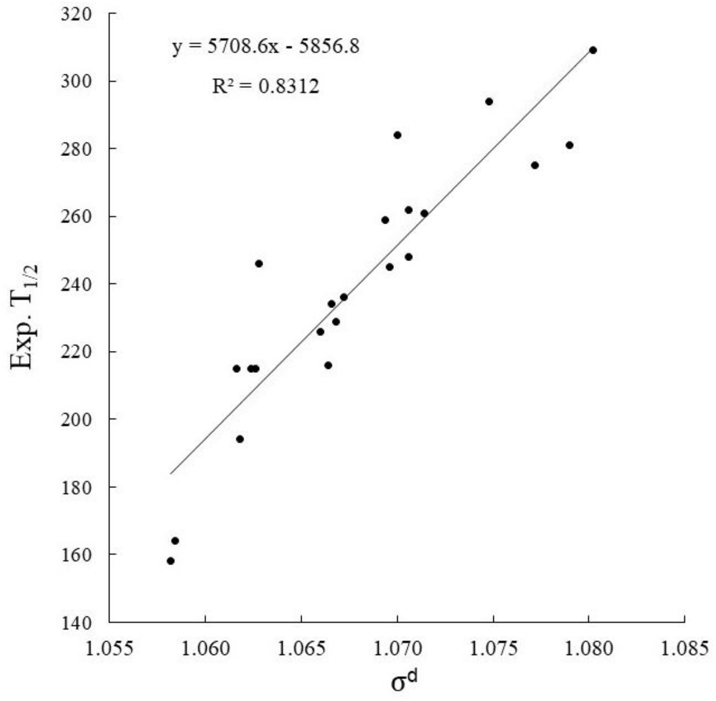

Figure S7: Linear relationship between the experimentally $T_{1/2}$ and the σ^d descriptor for the set of $[Fe^{II}(bpp^X)_2]^{2+}$ complexes obtained at the BP86/def2-TZVP level of theory.

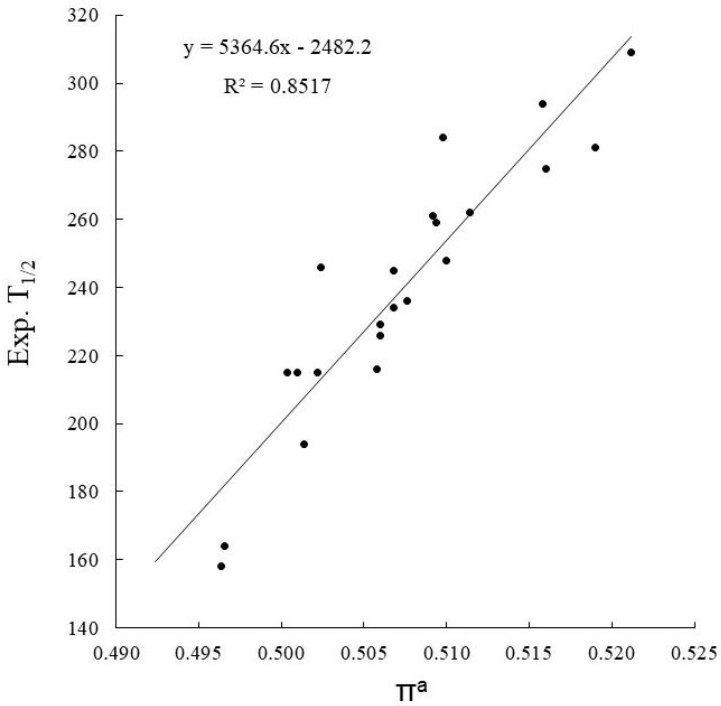

Figure S8: Linear relationship between the experimentally $T_{1/2}$ and the π^a descriptor for the set of $[Fe^{II}(bpp^X)_2]^{2+}$ complexes obtained at the BP86/def2-TZVP level of theory.

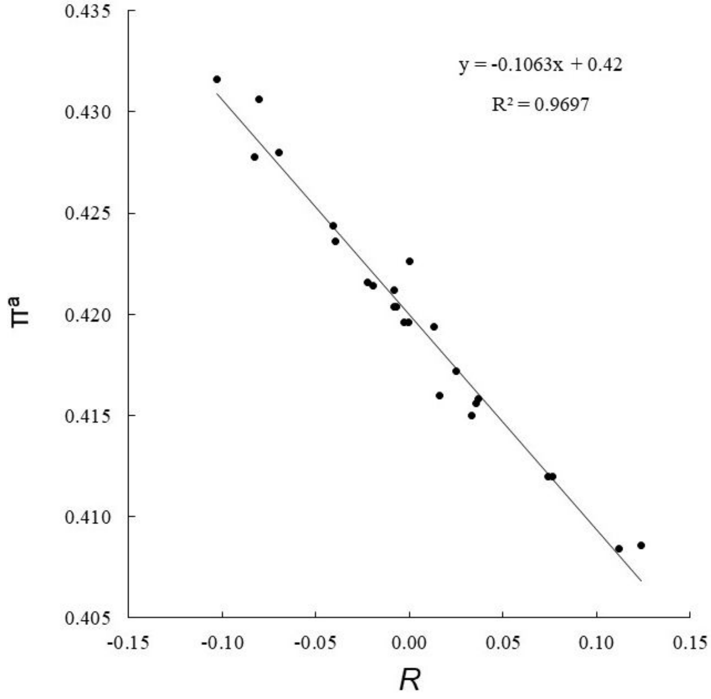

Figure S9: Relationship between free ligand resonance *R* and π^a descriptor for the set of $[Fe^{II}(bpp^X)_2]^{2+}$ complexes obtained at the TPSSh/def2-TZVP level of theory.

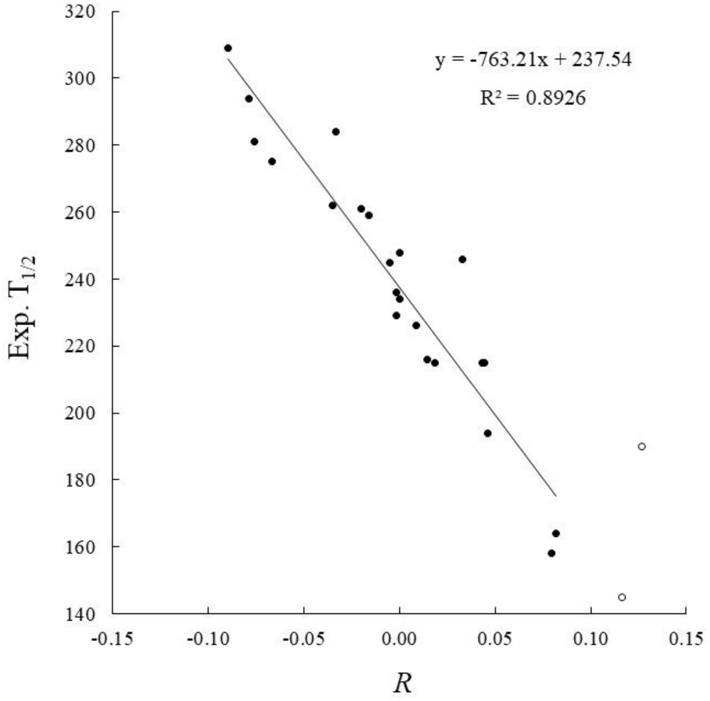

Figure S10: Linear relationship between the experimentally $T_{1/2}$ and the *R* descriptor in the different substituted *free* bpp^X ligands for the set of $[Fe^{II}(bpp^X)_2]^{2+}$ complexes obtained at the BP86/def2-TZVP level of theory. Empty circles (not used for the correlation) correspond to the upper limit values for X=NH_2 and X=NMe_2 (see text).

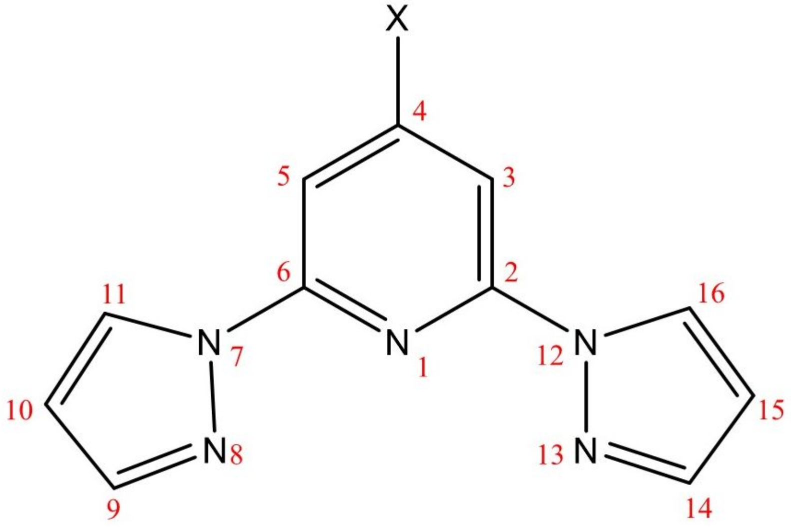

Figure S11: atomic numbering used to study the individual occupations in the *free* bpp^X ligands

Table S4: $2p_z$ EFOs occupations at all atoms in the *free* substituted bpp^X ligands obtained at the TPSSh/def2-TZVP level of theory.

X	1	2	3	4	5	6	7	8	9	10	11	12	13	14	15	16
H	1.2716	0.5904	0.8578	0.7922	0.8578	0.5904	1.4544	1.2082	0.6718	0.8774	0.7558	1.4544	1.2082	0.6718	0.8774	0.7558
CH_3	1.2668	0.5940	0.8612	0.7572	0.8612	0.5940	1.4508	1.2076	0.6776	0.8786	0.7548	1.4508	1.2076	0.6776	0.8786	0.7548
COOH	1.2522	0.5902	0.8254	0.8100	0.8318	0.5908	1.4532	1.2100	0.6734	0.8752	0.7544	1.4546	1.2104	0.6732	0.8746	0.7536
NH_2	1.2882	0.5990	0.8924	0.6842	0.8926	0.5990	1.4492	1.2076	0.6808	0.8812	0.7530	1.4494	1.2072	0.6800	0.8818	0.7536
NMe_2	1.2892	0.6020	0.8938	0.6842	0.8938	0.6020	1.4472	1.2072	0.6820	0.8838	0.7528	1.4472	1.2072	0.6820	0.8836	0.7530
NO_2	1.2530	0.5846	0.8280	0.7972	0.8280	0.5846	1.4572	1.2096	0.6692	0.8708	0.7544	1.4572	1.2096	0.6692	0.8708	0.7544
pz (pyrazol-1-yl)	1.2706	0.5934	0.8486	0.7254	0.8740	0.5944	1.4508	1.2088	0.6772	0.8784	0.7550	1.4530	1.2108	0.6758	0.8760	0.7526
OH	1.2834	0.5930	0.8934	0.6684	0.8746	0.5950	1.4522	1.2096	0.6762	0.8776	0.7540	1.4510	1.2074	0.6780	0.8788	0.7552
OMe	1.2810	0.5930	0.8880	0.6670	0.8728	0.6012	1.4514	1.2088	0.6798	0.8788	0.7522	1.4496	1.2076	0.6792	0.8794	0.7546
F	1.2832	0.5886	0.8754	0.6910	0.8754	0.5886	1.4568	1.2078	0.6690	0.8738	0.7564	1.4568	1.2078	0.6690	0.8738	0.7564
Cl	1.2760	0.5882	0.8616	0.8260	0.8616	0.5882	1.4568	1.2080	0.6688	0.8736	0.7562	1.4568	1.2080	0.6688	0.8736	0.7562
Br	1.2742	0.5882	0.8590	0.8532	0.8590	0.5882	1.4568	1.2080	0.6686	0.8736	0.7562	1.4568	1.2080	0.6686	0.8736	0.7562
I	1.2736	0.5882	0.8590	0.8634	0.8590	0.5882	1.4566	1.2080	0.6688	0.8738	0.7562	1.4566	1.2080	0.6688	0.8738	0.7562
SH	1.2740	0.5920	0.8686	0.8314	0.8654	0.5918	1.4522	1.2096	0.6754	0.8768	0.7542	1.4516	1.2090	0.6758	0.8772	0.7546
SCH_3	1.2738	0.5916	0.8700	0.8304	0.8658	0.5984	1.4510	1.2092	0.6794	0.8772	0.7532	1.4500	1.2078	0.6788	0.8792	0.7548
$SOCH_3$	1.2622	0.5928	0.8572	0.8304	0.8354	0.5848	1.4546	1.2114	0.6726	0.8738	0.7524	1.4526	1.2086	0.6736	0.8758	0.7560
$SOOCH_3$	1.2530	0.5870	0.8334	0.8434	0.8334	0.5870	1.4560	1.2096	0.6710	0.8724	0.7542	1.4560	1.2096	0.6710	0.8724	0.7542
cis-CH=CHPh	1.2624	0.5958	0.8564	0.7708	0.8426	0.5994	1.4512	1.2080	0.6796	0.8794	0.7522	1.4508	1.2072	0.6782	0.8784	0.7546
cis-CH=CH (C_6H_4CN-4)	1.2612	0.5924	0.8556	0.7742	0.8468	0.5938	1.4530	1.2092	0.6748	0.8752	0.7552	1.4524	1.2080	0.6740	0.8774	0.7548
cis-CH=CH ($C_6H_4NO_2$-4)	1.2604	0.5920	0.8550	0.7754	0.8466	0.5938	1.4532	1.2094	0.6744	0.8750	0.7552	1.4526	1.2080	0.6736	0.8770	0.7548
CH_2OH	1.2656	0.5922	0.8606	0.7682	0.8504	0.5936	1.4522	1.2098	0.6760	0.8764	0.7538	1.4510	1.2084	0.6788	0.8774	0.7540
SiPr	1.2742	0.5934	0.8662	0.8290	0.8632	0.5984	1.4508	1.2088	0.6796	0.8780	0.7540	1.4492	1.2074	0.6786	0.8794	0.7550
COOEt	1.2528	0.5914	0.8282	0.8050	0.8354	0.5916	1.4534	1.2088	0.6748	0.8758	0.7546	1.4548	1.2096	0.6740	0.8754	0.7530
$CSNHCH_3$	1.2566	0.5916	0.8572	0.7922	0.8298	0.5934	1.4540	1.2110	0.6738	0.8750	0.7524	1.4520	1.2084	0.6750	0.8774	0.7548

Table S5: $2p_z$ EFOs occupations at all atoms in the *free* substituted bpp^X ligands obtained at the BP86/def2-TZVP level of theory.

X	1	2	3	4	5	6	7	8	9	10	11	12	13	14	15	16
H	1.2474	0.6186	0.8594	0.7982	0.8594	0.6186	1.4314	1.1942	0.6974	0.8770	0.7762	1.4314	1.1942	0.6974	0.8770	0.7762
CH_3	1.2414	0.6226	0.8622	0.7606	0.8622	0.6226	1.4276	1.1938	0.7032	0.8800	0.7750	1.4276	1.1938	0.7032	0.8800	0.7750
COOH	1.2268	0.6192	0.8360	0.8098	0.8302	0.6182	1.4308	1.1954	0.6978	0.8760	0.7744	1.4298	1.1952	0.6982	0.8758	0.7760
NH_2	1.2630	0.6268	0.8944	0.6996	0.8948	0.6268	1.4260	1.1946	0.7062	0.8828	0.7736	1.4258	1.1950	0.7064	0.8830	0.7728
NMe_2	1.2630	0.6304	0.8948	0.7028	0.8948	0.6304	1.4240	1.1938	0.7094	0.8844	0.7730	1.4240	1.1938	0.7094	0.8844	0.7730
NO_2	1.2332	0.6138	0.8378	0.8026	0.8378	0.6138	1.4342	1.1944	0.6910	0.8702	0.7752	1.4342	1.1944	0.6910	0.8702	0.7752
pz (pyrazol-1-yl)	1.2452	0.6236	0.8760	0.7404	0.8522	0.6216	1.4296	1.1960	0.7020	0.8774	0.7714	1.4282	1.1942	0.7036	0.8788	0.7748
OH	1.2566	0.6242	0.8784	0.6780	0.8962	0.6226	1.4282	1.1936	0.7038	0.8802	0.7742	1.4290	1.1948	0.7030	0.8796	0.7730
OMe	1.2556	0.6294	0.8754	0.6794	0.8910	0.6212	1.4262	1.1950	0.7050	0.8814	0.7736	1.4278	1.1960	0.7052	0.8804	0.7724
F	1.2592	0.6172	0.8802	0.6974	0.8802	0.6172	1.4328	1.1944	0.6952	0.8750	0.7758	1.4328	1.1944	0.6952	0.8750	0.7758
Cl	1.2518	0.6172	0.8668	0.8278	0.8668	0.6172	1.4326	1.1946	0.6948	0.8748	0.7756	1.4326	1.1946	0.6948	0.8748	0.7756
Br	1.2518	0.6170	0.8634	0.8526	0.8634	0.6170	1.4326	1.1946	0.6946	0.8746	0.7754	1.4326	1.1946	0.6946	0.8746	0.7754
I	1.2498	0.6170	0.8632	0.8630	0.8632	0.6170	1.4324	1.1946	0.6948	0.8748	0.7754	1.4324	1.1946	0.6948	0.8748	0.7754
SH	1.2488	0.6218	0.8682	0.8336	0.8712	0.6220	1.4284	1.1944	0.7026	0.8794	0.7738	1.4290	1.1950	0.7022	0.8788	0.7732
SCH_3	1.2486	0.6288	0.8684	0.8338	0.8730	0.6220	1.4264	1.1952	0.7046	0.8812	0.7738	1.4272	1.1962	0.7044	0.8798	0.7722
$SOCH_3$	1.2370	0.6214	0.8590	0.8288	0.8392	0.6158	1.4308	1.1972	0.6982	0.8762	0.7714	1.4296	1.1940	0.7010	0.8760	0.7758
$SOOCH_3$	1.2296	0.6158	0.8358	0.8424	0.8358	0.6158	1.4318	1.1962	0.6964	0.8736	0.7740	1.4318	1.1962	0.6964	0.8736	0.7740
cis-CH=CHPh	1.2378	0.6276	0.8444	0.7752	0.8566	0.6242	1.4276	1.1934	0.7040	0.8796	0.7752	1.4288	1.1944	0.7046	0.8804	0.7722
cis-CH=CH (C_6H_4CN-4)	1.2358	0.6240	0.8484	0.7774	0.8566	0.6210	1.4288	1.1932	0.7012	0.8780	0.7752	1.4294	1.1944	0.7010	0.8774	0.7752
cis-CH=CH ($C_6H_4NO_2$-4)	1.2348	0.6238	0.8480	0.7788	0.8550	0.6214	1.4286	1.1934	0.7010	0.8768	0.7762	1.4296	1.1946	0.7006	0.8760	0.7760
CH_2OH	1.2404	0.6208	0.8616	0.7712	0.8514	0.6218	1.4292	1.1952	0.7026	0.8782	0.7730	1.4278	1.1958	0.7036	0.8794	0.7736
SiPr	1.2484	0.6244	0.8694	0.8318	0.8670	0.6278	1.4270	1.1958	0.7048	0.8806	0.7732	1.4256	1.1948	0.7050	0.8814	0.7738
COOEt	1.2280	0.6214	0.8370	0.8060	0.8306	0.6204	1.4306	1.1946	0.7004	0.8768	0.7730	1.4294	1.1940	0.7012	0.8774	0.7744
$CSNHCH_3$	1.2328	0.6206	0.8584	0.7940	0.8336	0.6216	1.4306	1.1960	0.7008	0.8770	0.7714	1.4292	1.1938	0.7026	0.8776	0.7750

Table S6: Experimental $T_{1/2}$, sigma-donor (σ^d), pi acceptor (π^a) and free ligand resonance (R) descriptors for substituted ligands in the $[Fe^{II}(pybox^X)_2]$ complexes. Values obtained at the BP86/def2-TZVP level of theory.

X	Exp $T_{1/2}$	σ^d	π^a	R
4-pyridyl	310	1.0794	0.5184	-0.0016
3-pyridyl	270	1.0774	0.5164	0.0076
2-thienyl	260	1.0708	0.5138	0.0254
3-thienyl	240	1.0718	0.5144	0.0294
Cl	270	1.0762	0.5172	0.0038
Br	280	1.0766	0.5176	-0.0026
H	260	1.0826	0.5152	0.0000
OMe	170	1.0634	0.5058	0.1016
Ph	240	1.0734	0.5158	0.0192
SMe	210	1.0680	0.5100	0.0662
N_3	215	1.0708	0.5124	0.0376
Me	220	1.0760	0.5156	0.0386

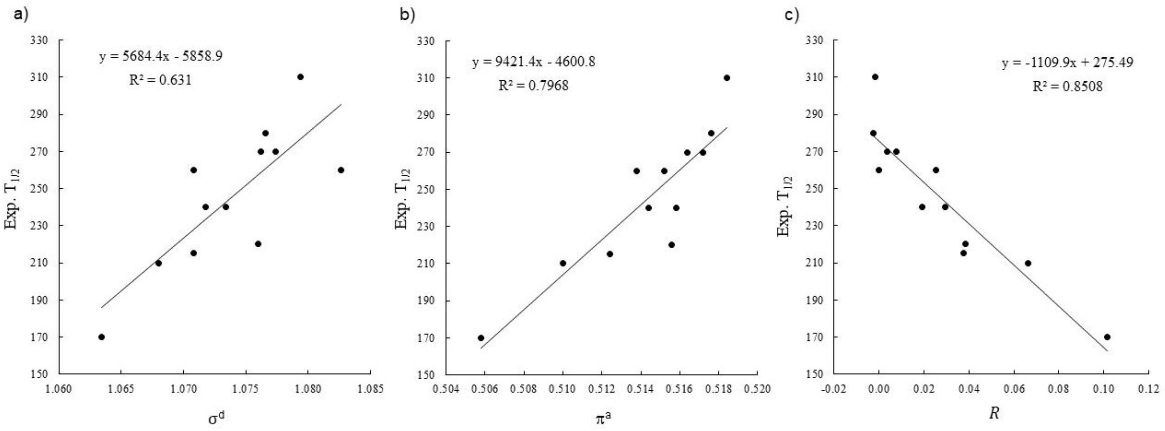

Figure S12: Linear relationship between the experimental $T_{1/2}$ values for the set of $[Fe^{II}(pybox^X)_2]^{2+}$ complexes and (a) σ^d descriptor for σ-donation, (b) π^a descriptor for π acceptor character, and (c) the R descriptor for the free ligands, obtained at the BP86/def2-TZVP level of theory.

Table S7: Experimental $T_{1/2}$, sigma-donor (σ^d), pi acceptor (π^a) and free ligand resonance (R) descriptors for substituted ligands in the $[Fe^{II}(pybox^X)_2]$ complexes. Values obtained at the TPSSh/def2-TZVP level of theory.

X	Exp $T_{1/2}$	σ^d	π^a	R
4Py	310	0.9384	0.4214	0.0002
3Py	270	0.9368	0.4196	0.0094
2Th	260	0.9336	0.4168	0.0312
3Th	240	0.9346	0.4172	0.0282
Cl	270	0.9358	0.4204	-0.0024
Br	280	0.9362	0.4210	-0.0084
H	260	0.9400	0.4202	0.0000
OMe	170	0.9264	0.4092	0.0992
Ph	240	0.9358	0.4186	0.0214
SMe	210	0.9312	0.4130	0.0590
N_3	215	0.9314	0.4152	0.0364
Me	220	0.9354	0.4186	0.0386
